# Manuelle Medizin, manuelle Therapie: Grundlagen, Wirkmechanismen, Indikationen und Evidenz

**DOI:** 10.1007/s00113-021-01004-8

**Published:** 2021-05-19

**Authors:** Hermann Locher

**Affiliations:** Zentrum für Orthopädie und Unfallchirurgie, Lindauerstr. 16, 88069 Tettnang, Deutschland

**Keywords:** Manuelle Medizin, Wirbelsäulenmanipulation, Wirbelsäulenmobilisation, Neurophysiologie, Schmerz, Manual medicine, Spinal manipulation, Spinal mobilization, Neurophysiology, Pain

## Abstract

Manuelle Medizin ist die medizinische Disziplin, die sich umfassend mit Diagnose, Therapie und Prävention reversibler Funktionsstörungen am Bewegungsorgan und anderen damit verbundenen Organsystemen befasst. Der Beitrag beleuchtet neuroanatomische und -physiologische Grundelemente der Wirkungsweisen manualmedizinischer Diagnostik und Therapie. Anhand neuester Literatur und Betrachtung verschiedener wissenschaftlicher Leitlinien wird die evidenzbasierte Wirksamkeit manualmedizinischer Verfahren dargestellt, im Einzelnen: akute und chronische Lumbalgie, zervikogener Kopfschmerz, Schulter- und Nackenschmerzen, radikulärer Armschmerz, dysfunktionelle thorakale Schmerzsyndrome, Erkrankungen der Rotatorenmanschette, Karpaltunnelsyndrom und Plantarfasziitis. Fallbeispiele veranschaulichen die klinische Vorgehensweise. Die Begrifflichkeit, die Provenienz und die klinische Präsenz der „Osteopathie“ werden ausführlich gewürdigt, und die nationalen und internationalen Vereinigungen und Gesellschaften der manuellen Medizin (Deutsche Gesellschaft für Manuelle Medizin [DGMM], European Scientific Society of Manual Medicine [ESSOMM], Fédération Internationale de Medicine Manuelle [FIMM]) werden lexikalisch dargestellt. Abschließend finden sich Kontraindikationen und ein Ausblick auf die Erfordernisse und Möglichkeiten der wissenschaftlichen Schmerzanalyse, wie sie in der Präambel der Leitlinie „Spezifischer Kreuzschmerz“ der Deutschen Gesellschaft für Orthopädie und Orthopädische Chirurgie (DGOOC) postuliert werden.

## Abstract (en)

Manual medicine is the medical discipline that comprehensively deals with the diagnosis, treatment and prevention of reversible functional disorders of the musculoskeletal system and other related organ systems. The article illustrates the neuroanatomical and neurophysiological basic elements and mechanisms of manual medical diagnostics and treatment. Based on the most recent literature and in consideration of various scientific guidelines, the evidence-based effectiveness of manual medical procedures is presented. In detail: acute and chronic low back pain, cervicogenic headache, neck and shoulder pain, radicular arm pain, dysfunctional thoracic pain syndromes, diseases of the rotator cuff, carpal tunnel syndrome and plantar fasciitis. Clinical case examples illustrate the clinical approach. The terminology, origin and clinical presence of “osteopathy” are described in detail and the national and international associations and societies of manual medicine, the German Society for Manual Medicine (DGMM), the European Scientific Society of Manual Medicine (ESSOMM) and the Fédération Internationale de Medicine Manuelle (FIMM) are lexically presented. Finally, contraindications for manual interventions and an outlook on requirements and possibilities of the scientific analysis of pain are presented, as they are postulated in the preamble of the guidelines on specific low back pain of the German Society for Orthopedics and Orthopedic Surgery (DGOOC).

Manuelle Medizin ist die medizinische Disziplin, die sich umfassend mit Diagnose, Therapie und Prävention reversibler Funktionsstörungen am Bewegungsorgan und an anderen damit verbundenen Organsystemen befasst. Das zielgerichtete Funktionieren verschiedener Systeme des menschlichen Körpers hängt von der sehr komplexen und fein abgestimmten Harmonie zwischen Afferenzen und Efferenzen auf segmentaler Ebene ab. Dieses segmentale Zusammenspiel steht unter intensiver Kontrolle durch absteigende Bahnen aus höheren Zentren (Stammhirn und alle darüber liegenden Gehirnanteile). Aufsteigende afferente Bahnen tragen Information als Rückkopplung im Sinne kybernetischer Systeme nach zentral [17]. Kommt es in einem oder mehreren Elementen dieses Zusammenspiels zu Sollwertverstellungen, treten initial reversible, später fixierte Funktionsstörungen und am Ende strukturelle Veränderungen auf. Zahlreiche klinische Symptome und Störungsbilder folgen diesem pathogenetischen Muster. In den Kursen zur Erlangung der Zusatzweiterbildung (ZWB) Manuelle Medizin wird dieser pathogenetischen Erkenntnisgewinnung große Bedeutung beigemessen [33].

Warum sind die Praxen der Manualmediziner und Manualtherapeuten so voll? Warum haben die gesetzlichen Krankenkassen 2019 8,7 Mrd. € nur für Heilmittel ausgegeben? Tendenz steigend (zum Vergleich Ärzte 40 Mrd., Arzneimittel 40 Mrd., Krankenhaus 80 Mrd. €, [11]). Warum werden Heilmittelrezepte in den orthopädischen/unfallchirurgischen Praxen mit einem Nachdruck angefordert, der seinesgleichen sucht? Wer kennt nicht die unerfreulichen Diskussionen um das Budget?

In dem sehr lesenswerten Buch *Die verlorene Kunst des Heilens* zitiert Lown (US-amerikanischer Kardiologe, geb. 1921, mit nicht weniger als 20 Ehrendoktoraten weltweit ausgezeichnet, [25]) Lewis, der in seinem Buch die *Jüngste Wissenschaft* [18, S. 45] schreibt: „Das Berühren ist das älteste und wirksamste Werkzeug des ärztlichen Handelns“. Lown führt aus: „Häufig ist das Gespräch beim ersten Interview recht unpersönlich. Die Beziehung zwischen Arzt und Patient ändert sich aber oft dramatisch nach der körperlichen Untersuchung“. Welche Disziplin untersucht heute noch intensiv klinisch (ganz)körperlich?

Die im Folgenden beschriebenen Beispiele sind aus der täglichen Praxis bekannt. Bei der Diagnose hilft kein bildgebendes Verfahren wirklich, und wenn überhaupt, erst in zweiter Linie. *Beachte*: Sie wird im Wesentlichen „manuell“ gestellt.

## Klinische Beispiele

- Sendet eine entzündete Appendix anhaltende Noziafferenzen zum Rückenmark, entsteht durch reflektorische Verschaltung ein Hypertonus der Bauchdecke (Abwehrspannung).
- Sendet ein lumbales Wirbelgelenk durch plötzliche Überlastung z. B. „Verheben“ oder Überrotation eine starke Noziafferenz zum Rückenmark, kommt es zu einer spastischen Anspannung der tiefen und mittleren Schichten der Wirbelsäulenmuskulatur, d. h., einer u. U. sehr schmerzhaften Bewegungseinschränkung im Segment (Blockierung im Sinne eines vertebralen Schutzreflexes, [19]).
- Führt eine zahnärztliche Behandlung mit konsekutiver Veränderung der Okklusion zu einer Fehlbelastung im Kiefergelenk, können über Afferenzen aus der Kaumuskulatur (N. mandibularis [unterer Ast des N. trigeminus, V_3_]) veränderte motorische Muster und Funktionsstörungen in der oberen HWS auftreten und z. B. Nacken- und Kopfschmerzen verursachen [12].
- Kommt es durch segmentale Funktionsstörungen in der unteren HWS zu minimalen zeitlichen Verzögerungen bei der Aktivierung des M. supraspinatus gegenüber dem Deltamuskel, entsteht am Anfang der Abduktion ein übermäßiger Druck auf die Supraspinatussehne mit entsprechenden Schmerzen (funktionelles Impingement, [3]). Hieraus können sich langfristig eine chronische Entzündung, Sehnendegeneration und Supraspinatusläsion/-ruptur entwickeln.

An diesen Funktionsstörungen setzt die manuelle Medizin an:

Die Hand des Therapeuten ist unter Anwendung geeigneter Techniken in der Lage, durch Erzeugung meist propriozeptiver Afferenzen aus verschiedensten Strukturen in die reflektorischen Regelkreise einzugreifen. Dabei werden schmerzinhibitorische Systeme aktiviert, und oft gelingt es, Regelkreise nozireaktiver Fehlregulation zu durchbrechen. In Ausnahmefällen gelingt auch die direkte Auflösung mechanischer Verklemmungen bestimmter gelenkig verbundener ossärer Funktionseinheiten. (*Beachte*: Dieser Mechanismus gilt nicht für die entzündete Appendix im ersten Beispiel.)

### Merke

Manuelle Medizin/manuelle Therapie zielt auf die Modulation systemrelevanter Afferenzen und Afferenzmuster zur Regulation dysfunktionaler Regelkreise.

## Neurophysiologische Hintergründe von Dysfunktion und Chronifizierung [21, 31]

Eine höchstrangige Runde von Experten aus dem Bereich der Grundlagenforschung (Neurophysiologie, Gehirnforschung, Neuroanatomie, Neuropharmakologie, myofasziale Schmerzforschung, Anästhesiologie und Algesiologie) hat auf Betreiben der Deutschen Gesellschaft für Manuelle Medizin (DGMM) und der Schweizerischen Ärztegesellschaft für Manuelle Medizin (SAMM) in einer Reihe von internationalen Symposien die Grundlagen manualmedizinischer Diagnostik und Therapie im Sinne translationaler Forschung erarbeitet und konsentiert [16, 23].

Die nachfolgenden Erklärungsversuche und Schaubilder wichtiger schmerzmedizinischer und funktioneller Begrifflichkeiten erreichen „einen an der Grenze der Tolerabilität liegenden Grad der Simplifizierung, bleiben aber im Grunde der Aussagen sehr nahe am wissenschaftlichen Wahrheitsgehalt und sind belastbar“ (Walter Zieglgänsberger pers. Mitteilung, emeritierter Direktor des Max-Planck-Institutes für Neuropharmakologie, München).

Der Autor des vorliegenden Beitrags hat diese Inhalte in das tägliche diagnostische und therapeutische Handeln internalisiert und erreicht damit eine fortgeschrittene Präzisierung der Differenzialdiagnosen und v. a. eine Verbesserung der differenzialtherapeutischen Detailplanung. Hierzu gehört auch und v. a. die Indikationsfindung für manualmedizinische Interventionen.

### Motorische Systemaktivierung

**Figure 1:**
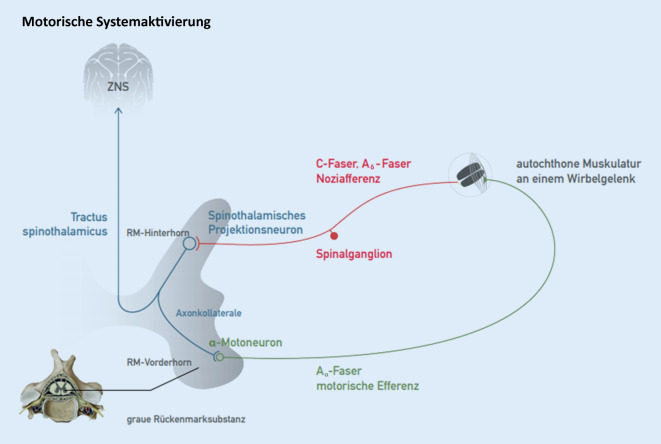

Der Organismus reagiert auf nozizeptive Reize über metamere und zentrale Verschaltungen im Sinne der nozireaktiven motorischen Systemaktivierung (Abb. 1).

Klinisch imponiert eine schmerzbedingte Störung der motorischen Koordination (z. B. abdominale Abwehrspannung, Extremitätenschutzreflex, Gangstörung bei z. B. aktivierter Koxarthrose, Fehlhaltung bei LWS-Blockierung, Zeichen der muskulären Dysbalance).

### Sympathische Systemaktvierung

**Figure 2:**
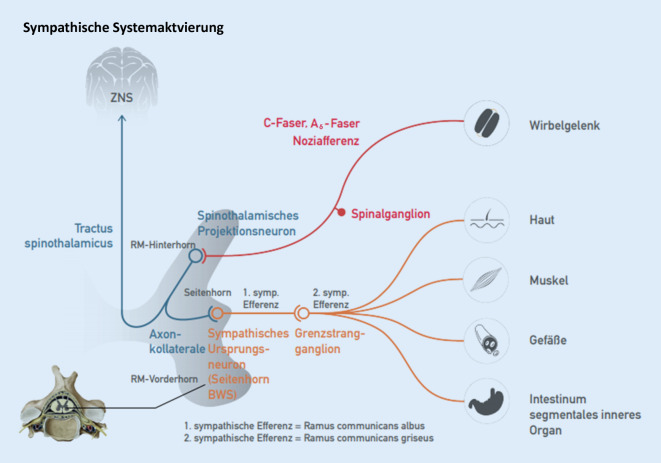

Axonkollateralen des Hinterhornneurons erregen auch sympathische Ursprungsneurone im thorakalen Seitenhorn und erzeugen vegetative Efferenzen (Abb. 2).

Klinisch können auftreten: Veränderungen der Hautdurchblutung, Piloarrektion, vermehrte Schweißsekretion, Tachykardie, Blutdrucksteigerung etc. Analog finden sich auch Wege der parasympathischen Dysregulation über den N. vagus und den N. pelvinus. Eine Extremform der sympathischen Systemaktivierung ist das „complex regional pain syndrome Typ I“ (CRPS Typ I, früher Sudeck-Dystrophie).

### Konvergenz

**Figure 3:**
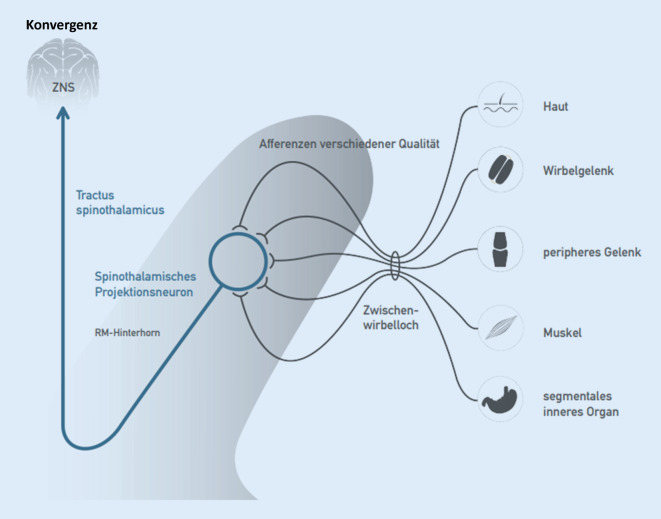

Am multirezeptiven Hinterhornneuron (MRH) konvergieren nicht nur Afferenzen aus den jeweiligen Wirbelgelenken, sondern auch Afferenzen aus verschiedensten anatomischen Systemen, die jeweils einem Segment zugeordnet sind, also Afferenzen aus Haut, Muskeln, Sehnen und inneren Organen (Abb. 3). Demnach können auch Noziafferenzen aus nichtvertebralen Strukturen in der gemeinsamen Endstrecke der motorischen Systemaktivierung zu vertebralen Dysfunktionen führen, wofür zahlreiche klinische Beispiele bekannt sind (Bronchialkarzinom, BWS-Blockierung, Adnexitis, LWS-Blockierung, Prostatitis, Sakroiliakalgelenk[SIG]-Blockierung u. v. a. m.) *Klinisches Cave*: Das erste Symptom eines Pankreaskarzinoms kann eine rezidivierende BWS-Blockierung sein.

### Periphere Sensibilisierung

**Figure 4:**
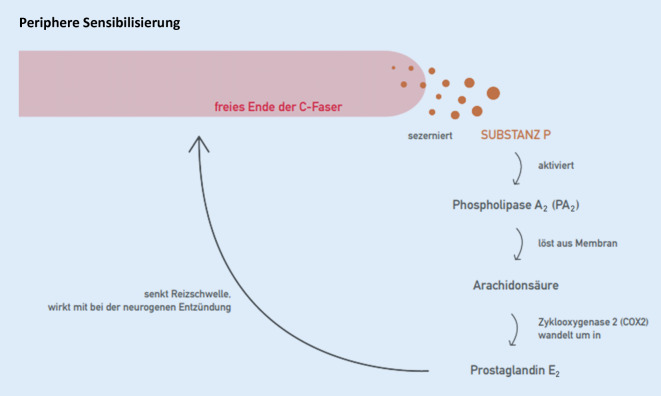

Wird ein Nozizeptor durch einen anhaltenden noxischen Reiz (z. B. UV-Strahlung, z. B. mechanische Überlastung eines Gelenks oder Wirbelgelenks oder Kompression von Nervi nervorum) länger dauernd überschwellig gereizt, verändert er sein biochemisches Verhalten (Abb. 4). Er sezerniert Neurokinine (Substanz P, „calcitonin-gene related peptide“ [CGRP] u. a.) in den Extrazellulärraum, die ihrerseits die sog. Entzündungskaskade anstoßen. Phospholipase A_2_ (PLA_2_) löst Arachidonsäure aus den Membranen; Zyklooxygenase 2 (COX 2) wandelt Arachidonsäure in Prostaglandin E_2_ um, das an Rezeptoren desselben Nozizeptors andockt und dort die Empfindlichkeit des Nerven erhöht. Dadurch wird die Reizschwelle des Nozizeptors abgesenkt; an der Haut wird Berührung schmerzhaft und am Gelenk wird Bewegung schmerzhaft. Die Neurokinine erzeugen auch Vasodilatation (Rubor) und Extravasation (Tumor) sowie neben der Absenkung der Reizschwelle (Dolor) und gelegentlich der Umwandlung proprio- in nozizeptive Afferenzen (Functio laesa) das Vollbild einer Entzündung, nämlich der neurogenen Entzündung. Im Fall einer neurogenen Entzündung wirken manualmedizinische Interventionen nicht: Hier ist vorbereitende Pharmakotherapie erforderlich.

*Klinisch-therapeutischer Seitenblick*: PLA_2_ wird gehemmt durch Steroide, COX 2 wird gehemmt durch nichtsteroidale Antirheumatika (NSAR).

### Zentrale Sensibilisierung, neurogene Neuroinflammation

**Figure 5:**
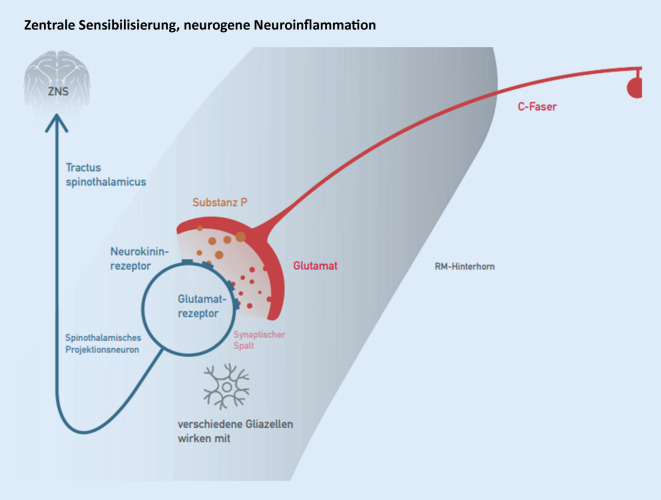

Auch im Rückenmark finden durch lang andauernden nozizeptiven Einstrom Sensibilisierungsprozesse statt, die im Wesentlichen mit den Vorgängen am Nozizeptor bei der peripheren Sensibilisierung vergleichbar sind (Abb. 5). Die Vorgänge im Rückenmark dürfen als wesentlich komplexer und komplizierter betrachtet werden, da auch im großen Umfang Mikroglia, Mastzellen, Astrozyten und neurovaskuläre Komplexe beteiligt sind. Ausgangspunkt dieser Vorgänge sind auch hier sekretorische Eigenleistungen der afferenten Fasern, und eine wesentliche Rolle spielt die zentrale nichtinduzierbare COX 2 bei der Synthese des ebenfalls zentral wirksamen Prostaglandin E_2_. Die heute gebräuchliche Begrifflichkeit für die Sensibilisierung des Rückenmarks lautet „neurogene Neuroinflammation“ nach Xanthos und Sandkühler [34].

*Klinische Bedeutung*: Noziafferenter Einstrom wird verstärkt fortgeleitet; schmerzinhibitorische Mechanismen werden abgeschwächt; nozizeptive rezeptive Felder vergrößern sich (pseudoradikuläre Ausstrahlungen, Head-Zonen, „referred pain“). Inhibitorische rezeptive Felder werden verkleinert, und die psychoaffektiven Komponenten der Schmerzwahrnehmung werden verstärkt: Ängste und dysphorische Zustände nehmen zu [1, 34].

Therapeutisch stehen zentral wirksame NSAR, Akupunktur und manualmedizinisch erzeugte geeignete Propriozeption im Vordergrund.

#### Merke

Mechanismen der peripheren und zentralen Sensibilisierung gelten als Chronifizierungsfaktoren und bestimmen wesentlich die klinische Symptomatik sowie differenzialdiagnostische und -therapeutische Erwägungen.

### Inhibitorische Systeme

**Figure 6:**
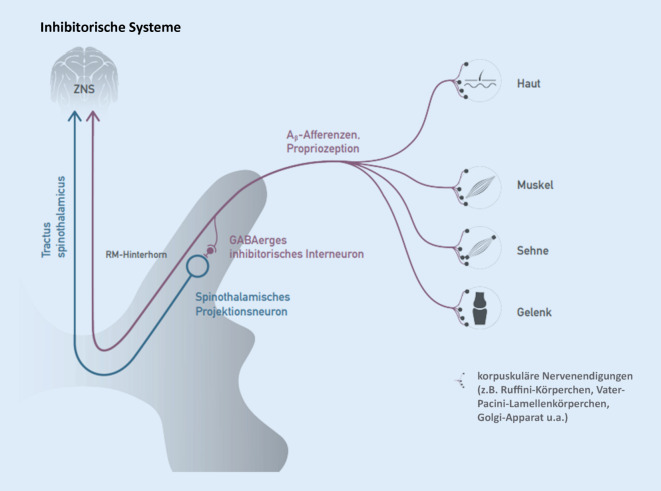

Neben den opioidergen und serotoninergen absteigenden inhibitorischen Funktionssystemen spielt im Hinblick auf die manuelle Medizin v. a. das GABAerge Hemmsystem eine wichtige Rolle (GABA: γ‑Aminobuttersäure, Abb. 6). Durch Erzeugung propriozeptiver Afferenzen (Berührung, Massage, Krankengymnastik, Bewegung im schmerzfreien Raum, Manipulation und Mobilisierung) werden schmerzhemmende Aktionspotenziale in GABAergen Interneuronen erzeugt, die das Aktivitätsniveau der multirezeptiven Hinterhornneurone reduzieren und damit die Durchschaltung nozizeptiver Erregungen abschwächen. Neben der Möglichkeit, mechanische Verklemmungen manuell zu lösen, dürfte diese manualmedizinische Möglichkeit des Eingreifens in neurophysiologische Schmerzregulation die noch bedeutendere Rolle bei der Erklärung manualmedizinischer Effekte spielen. Dies scheint nicht nur segmental zu wirken, da in einer entsprechenden Studie eine Anhebung der Druckschmerzschwelle auch an Orten fern der Manipulation nachgewiesen wurde [4]. Die Neurophysiologie der Schmerzhemmung i. Allg. ist schon sehr lange bekannt [33], findet aber erst in jüngerer Vergangenheit Eingang in die differenzialtherapeutische Therapieplanung [13, 22]. Klinisch zielen alle funktionellen Methoden auch auf die schmerzhemmenden Systeme.

#### Merke

Alle Formen der manuellen Therapie und viele andere funktionelle Therapiemethoden zielen u. a. auf die Beeinflussung schmerzinhibitorischer Systeme [23].

## Osteopathie

Es ist notwendig, im vorliegenden Kontext den Terminus „Osteopathie“ als Bestandteil und Erweiterung der manuellen Medizin [5] kritisch zu würdigen:

### Definitionen und Regelwerke

Für Begrifflichkeiten wie „Osteopathie“, „osteopathische Medizin“ und „osteopathische Behandlung“ fehlt eine klare weltweit akzeptierte Definition. In den USA existiert der Beruf „doctor of osteopathy“ (DO), der mit allen Rechten und Pflichten und nach der Absolvierung eines letztlich äquivalenten Studiums der Medizin dem des deutschen Arztes entspricht. Einen vergleichbaren Titel gibt es sonst weltweit nicht.

Osteopathie im Sinne der Zusammenführung diagnostischer und therapeutischer Methoden wird in verschiedenen Ländern Europas und auch weltweit von verschiedenen Berufsgruppen ausgeübt (Ärzte, Physiotherapeuten, Masseure, Heilpraktiker und auch Laientherapeuten). Ausbildungsgänge, Qualifikationen und Anwendungsstandards sind demnach hochgradig heterogen.

Die Bundesärztekammer betrachtet die „Osteopathie“ als Bestandteil und Erweiterung der manuellen Medizin und hat mit dem 2009 formulierten Gutachten „Wissenschaftliche Bewertung osteopathischer Verfahren“ [5] Standards für ärztliche Fortbildung und ärztliche Ausübung osteopathischer Verfahren gegeben. Osteopathische Medizin und osteopathische Verfahren werden von Fachärzten mit der ZWB Manuelle Medizin (Infobox 1) privatärztlich angewendet und privatärztlich abgerechnet. Sie sind in Deutschland nicht Bestandteil der kassenärztlichen Versorgung im Rahmen des Einheitlichen Bewertungsmaßstabs (EBM).

#### Infobox 1 Zusatzweiterbildung Manuelle Medizin nach der neuen Musterweiterbildungsordnung der Bundesärztekammer [7]

Die Musterweiterbildungsordnung (MWBO) der Bundesärztekammer (BÄK) formuliert für den Erhalt der Zusatzweiterbildung (ZWB) Manuelle Medizin Folgendes

*Definition*

Die ZWB Manuelle Medizin umfasst in Ergänzung zur Facharztkompetenz die Erkennung und Behandlung reversibler Funktionsstörungen des Bewegungssystems einschließlich ihrer Wechselwirkungen mit anderen Organsystemen mittels manueller Untersuchungs- und Behandlungstechniken

*Mindestanforderungen gemäß §* *11 MWBO*

- Facharztanerkennung in einem Gebiet der unmittelbaren Patientenversorgung
- 320 h Kursweiterbildung gemäß § 4, Absatz 8 in manueller Medizin
- oder 12 Monate Weiterbildung unter Befugnis an Weiterbildungsstätten (nur in wenigen Landesärztekammern so umgesetzt)

Zahlreiche Physiotherapeuten und Masseure wenden diagnostische und therapeutische osteopathische Techniken auf ärztliche Verordnung hin eigenverantwortlich an. Der Primärzugang zum Patienten (für Nichtärzte) setzt jedoch zwingend eine Berufserlaubnis als Heilpraktiker voraus.

### Wesen der Osteopathie

Der Begriff wurde von dem amerikanischen Arzt Andrew Taylor Still in der zweiten Hälfte des vorletzten Jahrhunderts in den Vereinigten Staaten in den Umraum der Heilkunst eingeführt. Die osteopathische Philosophie fußt auf folgenden 3 Prinzipien:

1. Alle verschiedenen Funktionssysteme innerhalb des Körpers sind eng miteinander verbunden und können nicht isoliert betrachtet werden.
2. Die Heilkunde hat sich ganz wesentlich auf die Selbstheilungskräfte des Organismus zu konzentrieren und diese zu fördern.
3. Diagnostisch und therapeutisch werden ausschließlich die Hände eingesetzt.

Die Begrifflichkeit „philosophy“ in Verbindung mit Heilkunde wird im Amerikanischen semantisch wesentlich enger gefasst verstanden als der Begriff „Philosophie“ in Europa. Die schulmedizinische Ärzteschaft steht dem Begriff „Philosophie der Osteopathie“ sehr zurückhaltend gegenüber. Die DGMM (Infobox 2) hat deshalb den Begriff der „philosophy“ durch „osteopathische Konzepte“ ersetzt, was mit der deutschen Facharztmedizin wesentlich kompatibler ist.

#### Infobox 2 Organisationen und wissenschaftliche Gesellschaften zur manuellen Medizin in Deutschland und international

Die Deutsche Gesellschaft für Manuelle Medizin (DGMM), Arbeitsgemeinschaft Wissenschaftlicher Medizinischer Fachgesellschaften (AWMF) Fachgesellschaft vertritt die manuelle Medizin wissenschaftlich, weiterbildungstechnisch und berufspolitisch im Kontext der Gestaltung und Weiterentwicklung innerhalb der medizinischen Versorgungsrealität.

*Mitgliedsgesellschaften der DGMM sind:*

- Dr. Karl-Sell-Ärzteseminar Neutrauchburg e. V. (MWE), Isny
- Deutsche Gesellschaft für Muskuloskeletale Medizin e. V. (DGMSM), Hamm, Boppard
- Ärztegesellschaft für Manuelle Medizin (ÄMM), Berlin

Die Mitgliedsgesellschaften der DGMM verstehen sich als gemeinnützige wissenschaftliche Gesellschaften zur Förderung von Wissenschaft, Lehre und Patientenversorgung im Umraum der manuellen Medizin und überblicken gemeinsam ca. 8000 Fachärztinnen und Fachärzte als ordentliche Mitglieder. Die DGMM ist damit der europa- und weltweit größte Zusammenschluss manualmedizinisch tätiger Ärztinnen und Ärzte.

*European Scientific Society of Manual Medicine (ESSOMM)*

Neben der Bundesrepublik Deutschland findet die manuelle Medizin aus ärztlicher Hand v. a. in der Schweiz und in Österreich Eingang in die medizinische Versorgung. Dort ist auch die ärztliche Qualifikation im Sinne einer Zusatzweiterbildung gesetzlich geregelt. In allen übrigen europäischen Ländern existieren verschiedene Qualitätskriterien und fließende Übergänge in der Anwendung zwischen Ärzten und anderen Therapeuten.

Im Jahr 2005 wurde die ESSOMM gegründet, um gemeinsame europäische Standards für die Inhalte und Weiterbildungscurricula der „additional competence in manual medicine“ (entspricht „Zusatzweiterbildung“) für Fachärzte festzulegen. In der ESSOMM sind heute 16 wissenschaftliche Fachgesellschaften aus 12 europäischen Nationen organisiert. Die ESSOMM vertritt ca. 14.000 europäische Fachärztinnen und Fachärzte, die manuelle Medizin anwenden.

*Union Européenne des Médecins Spécialistes (UEMS)*

Die UEMS bündelt alle wissenschaftlichen Fachgesellschaften Europas in „sections“ und fachübergreifende Aktivitäten in „multidisciplinary joint committees“. Sie legt die „European training requirements“ (ETR) für Facharztdisziplinen (z. B. „orthopedics and traumatology“, „obstetrics“, „dermatology“ etc.) und die ETR für „additional competences“ („manual medicine“, „sports medicine“, „algesiology“ etc.) fest. Die ETR „manual medicine“ wurden nach einem langjährigen Diskussions- und Harmonisierungsprozess von den Sektionen im Rahmen des UEMS Council in Larnaka, Zypern, 2015 verabschiedet und sind auf der UEMS-Homepage zu finden.

Damit hat die manuelle Medizin, ursprünglich im Bereich Naturheilkunde und Komplementärmedizin eingeordnet, den zweifelsfeien Status wissenschaftlicher Facharzt- und Universitätsmedizin.

Aufgrund der fehlenden Definition und der extremen Heterogenität aller Faktoren werden die „Osteopathen“ in weiten Teilen Europas von den Fachärzten (*beachte*: nicht jedoch von den Patienten) überwiegend kritisch und in weiten Kreisen durchaus ablehnend betrachtet.

### Terminologie

In der osteopathischen Terminologie werden im Wesentlichen 3 verschiedene Systeme beschrieben:

#### Parietales System.

Es umfasst Knochen, Gelenke, Faszien, Muskulatur und Bindegewebe sowie die zugehörigen peripheren Gefäße und Nerven.

#### Viszerales System.

Es umfasst die inneren Organe und ihre bindegewebigen Aufhängungen.

#### Kraniosakrales System.

Es umfasst zentralnervöse Strukturen, Schädelsuturen, Meningen und Rückenmarkhäute, basierend auf der Annahme spezifischer inhärenter Rhythmen des menschlichen Organismus.

Eine umfassende weltweite wissenschaftliche Literaturrecherche [5] konnte lediglich für den Bereich parietal eine befriedigende Evidenzlage aufzeigen. Der Begriff parietales System entspricht auch vollumfänglich dem, was den Inhalt des *Musterkursbuchs Manuelle Medizin* in der Weiterbildungsordnung der Bundesärztekammer abbildet [7]. Die Evidenzen für das viszerale System sind deutlich schwächer. Für den sog. kraniosakralen Rhythmus und die darauf aufgebauten Theorien fehlen bisher belastbare wissenschaftliche Grundlagen.

Nachdenklich darf stimmen, dass jüngst von einer Arbeitsgruppe um Messlinger in Erlangen Nervenfasern nachgewiesen worden sind, die von den Meningen im Schädelinneren durch die Schädelsuturen nach außen z. B. in den M. temporalis verlaufen [32]. Klinisch sind diese wahrscheinlich bei Kopfschmerzen und kraniomandibulärer Dysfunktion relevant.

*Und darüber hinaus* …

verfügen Osteopathen innerhalb der Gruppe der Heilberufe nach oft mehrjähriger Vollzeitfortbildung über eindrucksvolle Fähigkeiten im Sinne der integralen Beurteilung symptomatischer Funktionsstörungen und großes manuelles Geschick bei der therapeutischen Einflussnahme auf den Organismus, was ihre durchaus respektablen Erfolge in der Behandlung schmerzhafter Funktionsstörungen bei vielen Patienten nachvollziehbar macht.

Eine wirkliche Abgrenzung zur manuellen Medizin ist nicht möglich: Bezeichnenderweise heißt das führende Standardlehrbuch der US-amerikanischen Osteopathie: *Greenman’s Principles of Manual Medicine* [8].

#### Fazit

Osteopathie ist Bestandteil und Erweiterung der manuellen Medizin [5]**.**

## Behandlungstechniken [2, 35]

Im großen Kontext manuelle Medizin und Osteopathie kommen v. a. die folgenden Techniken zur Anwendung:

- Massage, Spezialmassage,
- axiale und vibrierende Traktion von Wirbelsäule und Gelenken,
- Mobilisation,
- Manipulation,
- Muskel-Energie-Techniken (postisometrische Relaxation), Muskeldehnung,
- „Strain“-/„Counterstrain“-Technik,
- „Myofascial-release“-Technik,
- viszerale Techniken.

## Behandelte Störungen und Krankheiten

Im Folgenden wird eine kleine Auswahl mit Bezug zu aktuellen Studien präsentiert.

### Spannungskopfschmerzen

Manuelle Therapien (Weichteiltechniken und Neuromobilisation) können die Häufigkeit, Dauer und Intensität von chronisch-rezidivierendem Spannungskopfschmerz signifikant vermindern, am besten in der Kombination beider Verfahren [10]. Bei der nichtmedikamentösen Therapie von chronischem Spannungskopfschmerz ergibt sich eine gute Evidenz für manuelle Therapien. Eine Behandlungsserie von 6 Behandlungen à 15–20 min erscheint ausreichend und damit auch ökonomisch.

### Zervikogener Kopfschmerz

In einer kleinen, aber sehr korrekt durchgeführten Studie [6] zeigten Verum- und Placebogruppe deutliche Verbesserungen der klinischen Symptome nach 3 Monaten manueller Therapie und schnitten besser ab als Kontrollgruppe. Die Arbeiten von Goadsby stellen die anatomischen und neurophysiologischen Grundlagen des zervikogenen Kopfschmerzes eindrücklich dar und erklären die manualtherapeutischen Effekte [12].

### Trigeminusneuralgiforme Schmerzzustände im Gesicht

Zervikotrigeminale Konvergenzen führen immer wieder zu neuralgiformen Schmerzen im Ausbreitungsgebiet des N. trigeminus und können durch manuelle Regulation der oberen HWS günstig beeinflusst werden [12].

### Nackenschmerzen, Zervikobrachialgien, Zervikodorsalgien

Eine randomisierte kontrollierte Studie mit 88 Probanden in 3 Gruppen [4] konnte nachweisen, dass sich die Griffstärke bei chronischen HWS-Armschmerzen nach einmaliger Manipulation signifikant verbessert. Der Schmerz war mit einer Behandlung nicht zu bessern, Zustände mit Zeichen der zentralen Sensitivierung (Schmerzausstrahlung nicht radikulär) bedürfen mehrmaliger Behandlungen im multimodalen Konzept.

Der positive Einfluss auf die Griffstärke zeigt jedoch die positive Einflussnahme auf das Phänomen der zentralen Sensitivierung und damit auch, wie zahlreiche persönliche Erfahrungen belegen, auf die Vorgänge der zentralen Schmerzverstärkung.

### Radikuläre Armschmerzen

Nach der S2k-Leitlinie „Zervikale Radikulopathie“ der AWMF (Reg.-Nr.: 030/082, [29]) soll nach bildgebendem und neurophysiologischem Ausschluss einer Nervenkompression mit motorischen Defiziten die Therapie multimodal konservativ erfolgen. Zu diesem Ansatz gehören neben Analgesie, Physiotherapie und Schmerzbewältigungstraining auch manuelle Techniken: Dabei können manuelle Traktionen und Mobilisationen der HWS nach dem Ausschluss von Kontraindikationen (Infobox 3) angewendet werden. Manipulationen mit Impuls sollen bei degenerativ bedingten zervikalen Radikulopathien nicht angewendet werden. Der Konsensus bestätigt die Erkenntnis, dass manuelle Techniken bei chronisch-degenerativen Erkrankungen nicht als Monotherapie, sondern im Wesentlichen als multimodale Kombinationsbehandlung über einen mittleren Zeitraum von einigen Wochen zum Einsatz kommen sollten.

#### Infobox 3 Kontraindikationen manualmedizinischer Interventionen

*Absolute Kontraindikationen*

- Frisches Trauma
- Osteoporotische Fraktur
- Bakterielle Entzündung
- Destruierender oder stabilitätsgefährdender Tumor
- Entzündliche Systemerkrankung im Schub
- Strukturelle Instabilität des Wirbelsäulensegments

*Relative Kontraindikationen*

- Osteoporose
- Schwere degenerative Veränderungen an der Wirbelsäule
- Hypermobilität
- Floride radikuläre Symptome
- Übermäßige passive Behandlungserwartung und fehlende Kooperation in einem komplexen Behandlungsschema mit der Notwendigkeit der Übernahme von Verantwortung durch den Patienten

### Thorax- und Rückenschmerzen

Akute BWS-Dysfunktionen und/oder Rippendysfunktionen können dramatische Schmerzzustände hervorrufen, die naturgemäß sehr angsteinflößend sein können, mit entsprechend emotionaler Schmerzverstärkung. Ein kurzfristiger Ausschluss internistischer Schmerzursachen ist zwingend notwendig (u. a. kardial, pulmonal oder abdominal). Bringt die manuelle Funktionsdiagnostik die Ursache an der BWS hervor, ist die manuelle Therapie mit Mobilisation oder Manipulation aussichtsreich [3].

#### Fallbeispiel: Wassersport

Ein 38-jähriger Französischlehrer stellt sich vor, wegen immer wieder an einer bestimmten Stelle der BWS auftretender Schmerzen, oft in Verbindung mit dem Gefühl der „Kreislaufschwäche“, ausstrahlend in den linken Thorax, gelegentlich mit Übelkeit und dem Gefühl von Herzarrhythmien. Er war kardiologisch, pulmologisch und abdominalchirurgisch mehrfach ohne jeglichen pathologischen Befund untersucht worden. Ein MRT der BWS ergab keinen pathologischen Befund. Physiotherapie, Trainingstherapie, Medikamente, Akupunktur und Hypnosebehandlung waren ohne Einfluss auf die Symptome, die sich immer wieder durch langes Sitzen erheblich verstärken.

Bei detaillierter Nachfrage nach dem Beginn der Schmerzen wird ein Unfall beim Wasserskifahren berichtet, bei dem er mit hoher Geschwindigkeit in das Wasser eingetaucht sei. Danach stellten sich über mehrere Tage Übelkeit und langsam zunehmende Schmerzen im mittleren Thorakalbereich ein, dann folgte ein schmerzfreies Intervall über mehrere Monate, weshalb der Zusammenhang nicht mehr gesehen wurde.

##### Klinisch.

Vollbild einer schmerzhaften Bewegungsstörung mit Hyperalgesiezeichen bei Th 6 ohne weitere orthopädische und neurologische pathologische Befunde.

##### Röntgenaufnahme der BWS.

Ohne pathologischen Befund.

##### Therapie.

Entscheidung zum Versuch einer manipulativen Remobilisierung, starkes Lösungsphänomen, völliges Verschwinden aller Symptome im Verlauf von einer Woche nach der Intervention.

##### Kommentar.

Klinisch relevante Dysfunktionen einzelner WS-Abschnitte können jahrelang persistieren und, wie im vorliegenden Fall, einer einmaligen manualmedizinischen Intervention zugänglich sein.

### Lumbale und lumbosakrale Dysfunktions- und Schmerzzustände

Für den akuten, nichtradikulären Lendenschmerz (sog. einfacher Hexenschuss) ergibt sich aus immer mehr qualitativ hochwertigen Untersuchungen, dass die manuelle Therapie (mit oder ohne Impuls) eine sinnvolle, effektive und nebenwirkungsarme Behandlung darstellt [28]. Dabei reicht zumeist eine Behandlung aus.

Die aktualisierte „Nationale VersorgungsLeitlinie Nicht-spezifischer Kreuzschmerz“ auf Basis einer systematischen Cochrane-Recherche spricht von einer „Kann“-Empfehlung für manuelle Therapie, Bewegungstherapie und Akupunktur. Alle anderen nichtmedikamentösen, nichtinvasiven Therapien werden ausdrücklich nicht empfohlen [6].

#### Leitlinie „Spezifischer Kreuzschmerz“ der Deutschen Gesellschaft für Orthopädie und Orthopädische Chirurgie [14]

Literaturrecherchen und Diskussionen bestätigen die in der ambulanten Praxis lange beobachtete Erfahrung, dass viele schmerzhafte Störungen des Haltungs- und Bewegungsorgans, darunter auch Funktionsstörungen der Kreuzdarmbeingelenke und chronische Kreuzschmerzen im multimodalen Assessment auf reversiblen Veränderungen basieren können sowie einem manuellen Diagnose- und Therapieansatz zugänglich sind.

#### Fallbeispiel: ein Fehltritt

##### Anamnese.

Eine 56-jährige Patientin (Untersuchung im Dezember 2020) berichtet über seit 6 Monaten bestehende, immer wiederkehrende belastungsabhängige, teils auch über längere Perioden (mehrere Wochen) andauernde Kreuzschmerzen, besonders beim Gehen, beim Stehen und unter Traglasten. Sitzen und Liegen gelingen problemlos. Bisher gelegentlich hatte sie mal kurz dauernde Kreuzschmerzen bei Überanstrengung, aber keine relevanten Begleiterkrankungen. In den letzten Wochen verspürte sie immer wieder Schmerzausstrahlungen in die rechtsseitige Oberschenkelaußenseite bis oberhalb des Kniegelenks.

„Beim Joggen am Sandstrand im Juli ist ein Bein im weichen Sand steckengeblieben, und es gab einen ruckartigen Zug am rechten Bein mit einem reißenden Schmerz rechts im Kreuz. Drei Tage starke Schmerzen, unter Ibuprofen Urlaub fortgesetzt, Kreuzschmerz seither nie mehr ganz weggegangen.“

##### Klinische Zeichen.

Straight Leg Raising Test (SLRT) und Bragard-Zeichen negativ; Sensibilität, Motorik, Reflexe unauffällig; rechtsseitig starke paravertebrale Druckdolenz L5/S1, Linksrotationsempfindlichkeit L5, Hauthyperalgesie in der Lumbosakralregion.

##### Röntgenaufnahmen der LWS in 2 Ebenen.

Diskrete Spondylosen L5 und S1, Unterbrechung der rechtsseitigen Interartikularportion L5, lumbosakrale Grad-1-Spondylarthrosen.

##### Diagnose.

Persistierende, belastungsabhängige Lumbalgie bei lumbosakraler Instabilität durch einseitige Spondylolyse L5 (rechts) und anhaltende lumbosakrale segmentale Dysfunktion nach axialem Traktion-Distorsion-Trauma*.*

##### Therapie.

Nach der Dehnung des M. quadratus lumborum und unspezifischer Rotationsmobilisierung der LWS erfolgt eine manipulative Rechtsrotation von L5. Es resultiert ein hör- und spürbares Lösungsphänomen mit anschließend subjektiver wesentlicher Besserung der Beweglichkeit. Nach 3 Tagen ist die Patientin anhaltend schmerzfrei (Rückkehr zum Zustand vor dem Joggen am Strand).

##### Kommentar.

Auch substanzielle strukturelle Veränderungen können anhaltende dysfunktionelle Schmerzzustände auf dem Boden der neuroregulativen Vorgänge hervorrufen, die der manuellen Therapie zugänglich sind. Das gilt auch für reflektorische Bewegungsstörungen bei Spondylarthrosen, Bandscheibenprotrusionen und Spinalstenosen, wobei nicht der Anspruch erhoben wird, die Grundkrankheit manuell erfolgreich behandeln zu wollen. Oft kann jedoch eine merkliche klinische und funktionelle Besserung der Beschwerden durch Beseitigung der begleitenden Dysfunktionen erreicht werden [27].

**Figure 7:**
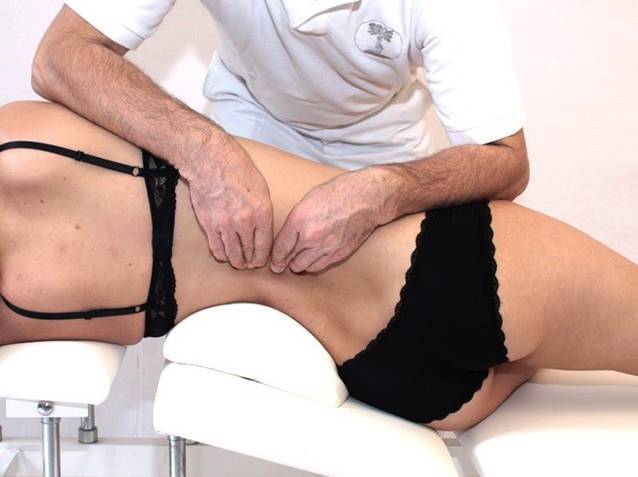

**Figure 8:**
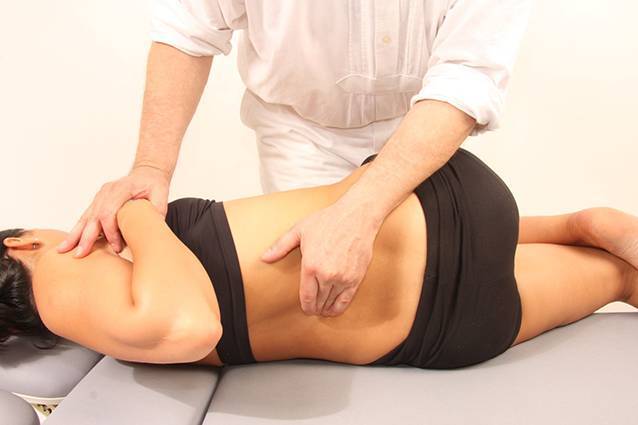

Detaillierte Anleitung und Bebilderung der gebräuchlichsten manuellen Techniken finden sich in [2, 35]; Abb. 7, 8).

##### Merke

Die Indikationsstellung zur manuellen Therapie folgt immer den Kriterien der „good clinical practice“ im Sinne vollumfänglicher fachärztlicher Verantwortlichkeit:

- detaillierte Anamnese,
- subtile klinische, funktionelle und neurologische Untersuchung,
- ggf. bildgebende und andere apparative Diagnostik,
- Würdigung psychosozialer Kontextfaktoren.

### Schmerzanalyse

Die Diagnosestellung bei schmerzhaften Störungen an WS und Extremitäten sollte immer den Regeln der systematischen Schmerzanalyse folgen:

1. Identifikation des Nozigenerators,
2. Benennung der motorischen, vegetativen und psychischen Reflexantwort,
3. Aktivierungsgrad der Chronifizierungsmechanismen,
4. Kondition der inhibitorischen Systeme

unter Betrachtung des Patienten im seinem psychosozialen Gesamtkontext [14, 20Präambel, 24].

### Bewegungseinschränkungen und -störungen der Extremitätengelenke

Bewegungseinschränkungen und schmerzhafte Bewegungsstörungen an Extremitätengelenken sind eine tägliche Herausforderung für alle mit dem Bewegungsorgan befassten Disziplinen. Derartige Veränderungen können posttraumatisch, -entzündlich und degenerativ auftreten und bedürfen der rehabilitativen Behandlung.

**Figure 9:**
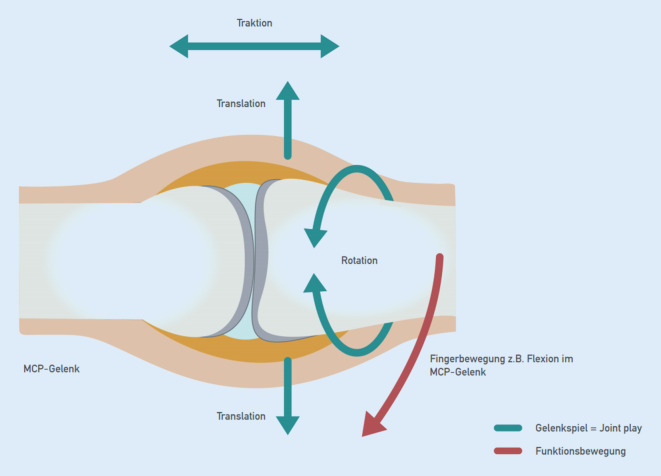

Pathogenetisch liegen Schrumpfungen der Gelenkkapsel, knöcherne Deformierung und reaktive Muskelverkürzung einzeln oder in Kombination zugrunde. Der forcierte Versuch, die *Funktionsbewegung* zu verbessern, führt zu Schmerzen und einer reaktiven Verstärkung der Bewegungseinschränkung. Die manuelle Therapie weicht deshalb auf das Feld der *Gelenkspielbewegungen* („joint play movements“) aus und sucht über einen Zugewinn an Beweglichkeit im Gelenkspiel die atraumatische Besserung im Raum der Funktionsbewegungen (Abb. 9).

**Figure 10:**
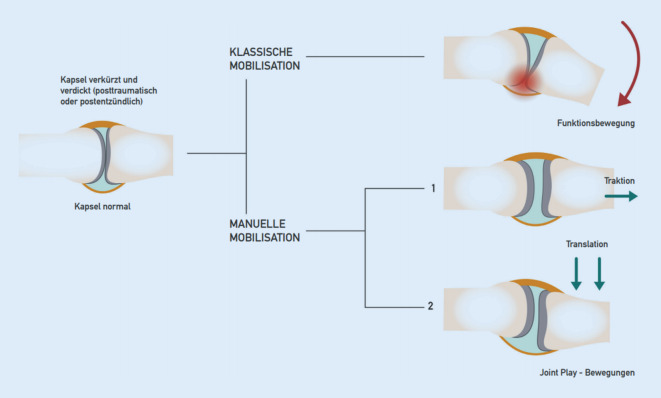

Mobilisierungen im Raum der Gelenkspielbewegungen erlauben eine Erweiterung des gesamten Bewegungsraumes eines Gelenks und bewirken eine Verbesserung der Funktionsbewegung ohne Stimulation von nozizeptiven Afferenzen (Abb. 10).

Darüber hinaus bewirkt eine gelenkspaltnahe, mobilisierende Bewegung mit 1 Hz die:

- Detonisierung der Muskulatur,
- bessere Knorpelernährung,
- Vermehrung der Synovialflüssigkeit,
- Beruhigung der Nozizeption,
- Verminderung der Noziafferenz,
- Erweiterung der Joint-play-Räume,
- konsekutive Verbesserung der Funktionsbewegung,
- Induktion einer „long-term depression“ (schmerzinhibitorischer Mechanismus an Rückenmarkneuronen) des Zentralnervensystems (ZNS; [34]).

#### Merke

Durch manualmedizinische Erweiterung der Gelenkspielbewegungen (Joint play movements) können erhebliche Zuwächse im Bereich der Funktionsbewegungen ohne Zunahme von Schmerz und Verspannung erreicht werden. Manuelle Medizin ist damit ein essenzieller Faktor in der posttraumatischen und -operativen Rehabilitation.

### Schulterschmerzen

Bei den Rotatorenmanschettensyndromen war die lokale manuelle Mobilisation mit Bewegungstherapie den anderen Therapien (medikamentös, Elektrotherapien, Kinesio-Tape, spinale Manipulation) signifikant überlegen [15]. Zusammengefasst: Die Metaanalyse aller systematischen Reviews und „randomized controlled trials“ (RCT) für 5 in der Praxis häufige Schultererkrankungen ergab die höchste relative Evidenz („moderat bis gut“) für wiederholte manuelle Therapie bei Supraspinatus-Impingement-Diagnosen [26].

### Karpaltunnelsyndrom

*Randomized controlled trial*: 100 Patienten, Beschwerden länger als 6 Monate, Hypästhesie, Hoffmann-Tinel- und Phalen-Zeichen 3‑fach positiv. Gruppe 1: 50 Patienten mit klassischer Operation (Spaltung des Lig. carpi transversum), Gruppe 2: 50 Patienten mit einmal wöchentlicher manueller Therapie für 30 min.

*Ergebnis nach 12 Monaten*: Beide Gruppen waren in allen Parametern signifikant besser als die Baseline, kein signifikanter Unterschied zwischen den Gruppen beim Schmerz, keine Unterschiede der Temperaturempfindlichkeit in beiden Gruppen.

*Fazit*: Eine konservative Therapie des Karpaltunnelsyndroms mithilfe der manuellen Therapie ist der Operation ebenbürtig [9].

### Plantarfasziopathie

In einem kritischen Review aller RCT mit Evaluation ihrer Qualität wurden 6 RCT hoher Qualität identifiziert, die in einer Metaanalyse zusammengeführt wurden. Die besten Ergebnisse wurden mit einer sehr festen Weichteilbehandlung als myofasziale Entspannung speziell der Wade und der Plantarfaszie erreicht, wobei auch lokale Schmerzpunkte in der Muskulatur durch Druck bis zum Verschwinden behandelt wurden. Gelenkmobilisationen waren weniger effektiv. Plantarer Fersenschmerz kann manuell erfolgreich behandelt werden, wenn der funktionell zusammenhängende Bereich der Wadenmuskulatur und ihrer Faszie zusammen mit der Plantarfaszie durch eine manuelle Druckbehandlung entspannt wird („fasziale Release-Technik“, [30]).

## Fazit für die Praxis

- Die Verfahren der manuellen Medizin gehören zu den evidenzbasiert wirksamen Therapieformen bei Funktionsstörungen und Schmerzen an Wirbelsäule und Extremitäten.
- Die Anwendung von manueller Medizin folgt allen Regeln der „good clinical practice“ (Anamnese, klinische, ggf. bildgebende Untersuchung, ggf. Labor- und Spezialuntersuchungen).
- Die Anwendung von manueller Medizin ist nur in den Händen von speziell weitergebildeten FachärztInnen und entsprechend weitergebildeten TherapeutInnen effizient und sicher.
- Manuelle Medizin darf nur unter Beachtung aller differenzialdiagnostischen Erwägungen und der Kontraindikationen für manuelle Medizin angewendet werden.
- Korrekt eingesetzte manuelle Medizin hat eine hohe Compliance unter den Patienten und ist in allen Fächern, die mit dem Bewegungsorgan befasst sind, unverzichtbarer Bestandteil des diagnostischen und therapeutischen Portfolios.
